# Ketoconazole-Loaded Mucoadhesive Nanoemulsions for the Better Management of Topical Fungal Infections: Optimization, In Vitro, Ex Vivo, and In Vivo Assessments

**DOI:** 10.3390/pharmaceutics18050612

**Published:** 2026-05-17

**Authors:** Mashan Almutairi, Ahmed Adel Ali Youssef, Gehad M. Subaiea, Ahmed Alobaida, Sultan Almuntashiri

**Affiliations:** 1 Department of Pharmaceutics, College of Pharmacy, University of Ha’il, Ha’il 81442, Saudi Arabia; a.alobaida@uoh.edu.sa; 2 Department of Pharmaceutics & Pharmaceutical Technology, Faculty of Pharmacy, Kafrelsheikh University, Kafrelsheikh 33516, Egypt; eldiasti89@pharm.kfs.edu.eg; 3 Department of Pharmacology and Toxicology, College of Pharmacy, University of Ha’il, Ha’il 81442, Saudi Arabia; g.subaiea@uoh.edu.sa; 4 Department of Clinical Pharmacy, College of Pharmacy, University of Ha’il, Ha’il 81442, Saudi Arabia; s.almuntashiri@uoh.edu.sa

**Keywords:** ketoconazole, nanoemulsion, fungal, in vivo, flux, ex vivo, irritation, permeation

## Abstract

**Background/Objective:** The introduction of Ketoconazole (KZ, Nizoral^®^) in 1977 by Janssen Pharmaceutica marked a significant milestone in medical mycology as the first broad-spectrum oral antifungal agent. However, KZ is a highly lipophilic compound, presenting significant challenges in the development of efficient topical formulations. Moreover, oral KZ has undergone labeling revisions and market withdrawal due to serious hepatic side effects. This study aimed to design, optimize, and evaluate KZ-loaded nanoemulsions (NEs; KZ-NEs) as a delivery platform that could improve skin bioavailability and antifungal activity. **Methods:** Optimized KZ-NEs were converted to a mucoadhesive formulation (KZ-NEC) by the addition of Carbopol^®^ 940 NF to enhance the adherence of the formulations to the skin surface. NEs were evaluated concerning physical appearance, globule size, polydispersity index, zeta potential, pH, viscosity, and drug content. Optimized KZ-NE and lead KZ-NEC formulations were further evaluated for in vitro release, ex vivo skin permeation and deposition, skin irritation, and in vivo studies. **Results:** In vitro release studies revealed that nanocarrier systems provided a sustained release of KZ over 24 h. The ex vivo permeability coefficients of KZ from the optimized KZ-NE and lead KZ-NEC formulations were approximately four- and three-fold greater than that achieved with the marketed cream formulation, respectively. In addition, the C_max_ of the lead KZ-NEC formulation (14.4 ± 1.1 μg/mL) was significantly higher (*p* < 0.05) compared with the marketed cream formulation (10.5 ± 0.5 μg/mL). Moreover, in vitro antifungal susceptibility testing showed that KZ demonstrated improved antifungal efficacy when incorporated into the KZ-NE and KZ-NEC formulations. Neither of the NE-based formulations caused any alterations in skin color or morphology during the 24 h visual observation period. Both NE-based formulations were stable for 90 days (the last time-point tested) at three different storage conditions. **Conclusions:** NE-based formulation could serve as an effective topical delivery platform for KZ and could improve therapeutic outcomes for patients with topical fungal infections.

## 1. Introduction

Fungal infections can be superficial, systemic, or both. However, superficial fungal infections (SFIs) are among the most frequent human infectious diseases encountered in clinical practice [1]. SFI is caused by pathogenic fungi and is limited to hair, nails, epidermis, and mucosa [1]. Although these SFIs are rarely dangerous or life-threatening, they are critical because of their common worldwide distribution and person-to-person transmission through fomites. SFIs are thought to impact 20 to 25% of the world’s population, and the rate of incidence continues to rise worldwide [2,3]. Treatment of SFIs is mainly dependent on the use of azole antifungals (imidazole/triazole) and/or allylamine antifungals, applied topically in a short treatment course or administered orally for longer periods, depending on the fungal infection location and severity [4].

The introduction of Ketoconazole (KZ, Nizoral^®^) in 1977 by Janssen Pharmaceutica marked a significant milestone in medical mycology as the first broad-spectrum oral antifungal agent. In July 1981, KZ received approval from the FDA for the treatment of systemic fungal infections. For nearly a decade thereafter, it remained the sole oral antifungal option available for managing systemic mycoses [5]. Oral KZ has undergone labeling revisions and market withdrawal due to serious hepatic side effects [5]. However, topical KZ remains widely regarded as both effective and safe for managing superficial fungal infections. Furthermore, emerging dermatologic applications for topical KZ have been identified, including its use in conditions such as onychomycosis, blepharitis, and certain forms of alopecia [6].

KZ is a broad-spectrum imidazole antifungal agent that exerts its activity by disrupting ergosterol biosynthesis through inhibition of a cytochrome P450-dependent enzyme, thereby altering the structural integrity and functional properties of the fungal cell membrane, consistent with the mechanism of other imidazole derivatives. Early in vitro investigations demonstrated KZ’s efficacy against a wide range of organisms, including dermatophytes, yeasts, molds, and dimorphic fungi, as well as certain bacterial species. Additionally, in vivo studies confirmed its therapeutic potential in animal models of oral, vaginal, cutaneous, and systemic candidiasis [7,8,9,10].

According to the Biopharmaceutical Classification System (BCS), KZ is categorized as a BCS class II drug with low aqueous solubility and high permeability [11]. KZ is a highly lipophilic compound (log *p* = 4.74) with poor aqueous solubility (0.04 mg/mL), presenting significant challenges in the development of efficient topical formulations capable of achieving optimal therapeutic efficacy across the stratum corneum barrier [12]. KZ is pharmaceutically marketed as shampoo and cream drug products. Creams are often associated with poor drug penetration to the target site, adverse effects such as swelling, irritation, erythema, pruritus, and contact dermatitis, and inadequate therapeutic efficacy for managing deeper skin infections [12,13]. Likewise, the primary disadvantages of applying drugs in a shampoo formulation are the limited contact time for the drug to be absorbed and a higher risk of localized side effects like skin irritation and dryness. Therefore, improved permeation at the site of fungal infection and a slow-controlled release profile to manage these issues is desired for KZ topical application.

Nanoparticulate formulations encapsulating antifungal agents improve the penetration of these actives across the skin; therefore, these nanoparticles eliminate the undesirable adverse effects of the drugs encountered during systemic application [1]. Nanoemulsions (NEs) are thermodynamically stable, isotropic systems characterized by globule sizes typically ranging from 20 to 200 nm [14,15]. NEs exhibit countless advantages over macroemulsions, such as increased stability, a massive increase in the interfacial area, quick absorption by internalization into the epithelial cells of barrier membranes through receptor-mediated endocytosis, and the ability to improve drug solubility and, henceforth, drug penetration [14,16]. NEs can also be prepared without the use of organic solvents [14,16]. In addition, NEs offer a high solubilization capacity for both hydrophilic and hydrophobic drugs, owing to the surfactant-stabilized interface formed between the dispersed oil and aqueous phases [16]. Furthermore, the surface charge, as well as small globule size, provides outstanding kinetic stability against sedimentation and flocculation to resist the process of coalescence (creaming) of oil globules during formulation storage since the Brownian motion of these small globules overcomes the force of gravity [14,16]. NE formulations have been reported as a topical formulation to deliver different drugs for the treatment of several dermatological diseases.

Topical drug delivery is the preferred route for the treatment of various skin disorders. The topical route has several advantages compared to the conventional oral and parenteral routes, which include high efficacy with less frequent dosing to a specific site through direct penetration of skin layers, avoiding first-pass metabolism, improving pharmacological response and patient compliance (noninvasive), and ease of application along with the possibility of terminating therapy whenever necessary [15,17]. For the treatment of SFIs, the delivery of antifungal agents is limited by the barrier nature of the stratum corneum layer of the skin and drug solubility. NE could provide a better option for the topical application of low aqueous solubility drugs such as KZ by incorporating the drug into an O/W emulsion. In the current study, topical O/W KZ-loaded NEs were prepared after screening various oils to improve the drug solubility and enhance skin permeability. Optimal production parameters, such as globule size and zeta potential, were adjusted using a full factorial design. The optimized KZ-NE formulation was converted to KZ-NEC by the addition of Carbopol^®^ 940 NF as a gelling agent. Further, optimized KZ-NE and lead KZ-NEC formulations were evaluated for stability, ex vivo skin permeation and deposition, antifungal activity testing, in vivo skin irritation studies, and in vivo evaluation against the marketed cream formulation as a control in an animal model.

In contrast to previously reported topical KZ formulations, the present study introduces a novel approach by combining NE technology with a mucoadhesive delivery system using Carbopol^®^ 940 NF to enhance both drug retention at the site of application and transdermal delivery. Furthermore, systematic optimization was carried out using a full factorial design to precisely control critical formulation parameters. Unlike most earlier studies, which primarily focus on in vitro or ex vivo evaluation, this work provides a comprehensive assessment through the integration of in vitro release, ex vivo permeation, and in vivo dermatokinetic studies. In addition, the developed formulations were directly compared with a marketed Ketoconazole cream, highlighting their potential clinical relevance. Collectively, this combined strategy represents an advancement over existing KZ topical nanoformulations by offering improved performance and a more rigorous evaluation framework.

## 2. Materials and Methods

### 2.1. Materials

KZ was purchased from TCI^®^ chemicals (Tokyo Chemical Industry, Portland, OR, USA). Polyethylene glycol 200 (PEG 200) was purchased from Sigma-Aldrich (St. Louis, MO, USA). PEG 400 was purchased from BASF Corporation (Florham Park, NJ, USA). Carbopol^®^ 940 NF grade was acquired from Spectrum Chemicals (New Brunswick, NJ, USA). Tween^®^ 80, Oleic acid, and Ultra-Fast liquid chromatography (UFLC) grade solvents were obtained from Fischer Scientific (Hampton, NH, USA). All screened oils were purchased from Gattefossé India (Mumbai, Maharashtra, India). Glassware such as scintillation vials, centrifuge tubes, and UFLC vials was acquired from Fischer Scientific (Hampton, NH, USA).

#### Animals

Male Wistar albino rats (*Rattus norvegicus*), weighing between 190 and 250 g, were purchased from the laboratory animal colony of the Egyptian Organization of Biological Products and Vaccines (VACSERA, Helwan, Cairo, Egypt). All rats were maintained under optimal hygienic conditions, provided with appropriate feed, and granted unrestricted access to water. All animal experiments were conducted in accordance with protocols approved by the Institutional Animal Care and Use Committee of Kafrelsheikh University (license number: KFS-IACUC/179/2024). The study protocol was reviewed and approved by the Faculty of Pharmacy Ethics Committee to ensure adherence to the Guide for the Care and Use of Laboratory Animals, published by the U.S. NIH (Publication No. 85-23, revised 2011).

### 2.2. Methods

#### 2.2.1. Analytical Method

Samples were analyzed for KZ using a previously reported HPLC method with slight modifications [18]. Quantification of KZ was performed using a Shimadzu UFLC system equipped with a SIL-20AC autosampler, an SPD-M20A UV/VIS photodiode array detector, and an LC-20AD solvent delivery module (Shimadzu Corporation, Nakagyo-Ku, Kyoto, Japan). Separation was carried out using reversed-phase chromatography on a Waters Symmetry^®^ C18 column (5 μm, 150 × 4.6 mm, Waters, Milford, CA, USA). The mobile phase consisted of acetonitrile and 0.2% triethylamine (45:55 *v*/*v*), adjusted to pH 6.4 with orthophosphoric acid, and delivered at a flow rate of 1.0 mL/min. Before use, the mobile phase was degassed and filtered through a 0.45 μm membrane filter (MF-Millipore™, Saint-Quentin, Yvelines, France). Detection was performed at 230 nm with a sensitivity setting of 1.0 AUFS (Absorbance Units Full Scale). The injection volume was 20 μL, and the column temperature was maintained at 25 °C.

#### 2.2.2. Screening Oils

The solubility of KZ in various oils was screened by adding 5 mg of KZ to 1 g of oil under continuous magnetic (2000 rpm) stirring at 80 ± 2 °C in separate 3 mL glass vials. The mixtures were observed to determine whether the drug was dissolved completely or not. If solubility was achieved, the KZ amount was increased in 5 mg increments, repeating the mixing and observation process after each addition. This stepwise procedure continued until the maximum amount of KZ that could be solubilized in 1 g of oil was added [14].

#### 2.2.3. Saturation Solubility in the Candidate Oil

To evaluate the solubility of KZ in the selected oil, an excess amount of the drug (300 mg) was added to 1 g of oil in glass vials, which were then placed in a reciprocating water bath (Precision™, Waltham, MA, USA). The mixture was maintained at 25 ± 0.5 °C with agitation at 100 rpm for 48 h. After equilibration, the KZ–oil mixtures were centrifuged at 13,000 rpm for 20 min (Fisherbrand™ AccuSpin 17R). The resulting supernatant was collected, filtered through a 0.22 μm nylon membrane, and analyzed for KZ concentration by UFLC following appropriate dilution with methanol to bring the KZ concentration within the linear range of the validated UFLC method before injection [19].

### 2.3. Experimental Design

Based on the literature review, the most important independent variables (factors) that can influence the physical properties of the NEs were identified and selected [20,21]. A 2^3^-factorial design [8 experimental runs; design generated using DesignExpert^®^ software (8.0.7.1, Minneapolis, MN, USA)] was employed to develop the KZ-NE formulations, where three independent variables were applied to determine the optimal levels for the mean globule size (GS, nm, Y_1_) and (ZP, Y_2_) at 2 levels assigned by the design. The selected independent variables were the oil concentration (Oil, % *w*/*v*, A), lipophilic surfactant concentration (Span^®^ 80, % *w*/*v*, B), and hydrophilic surfactant concentration (Tween^®^ 80, % *w*/*v*, C) which were studied at two different levels: level +1 (A: 5.0% *w*/*v*, B: 1.0% *w*/*v*, and C: 3.0% *w*/*v*) and level −1 (A: 3.0% *w*/*v*, B: 0.5% *w*/*v*, and C: 2.0% *w*/*v*). Table 1 and Table 2 present the details on the experimental 2^3^ factorial design.

#### 2.3.1. Preparation KZ-NEs

Oil-in-water (O/W) type KZ-NE formulations were prepared by homogenization coupled with the probe sonication method [14]. Briefly, KZ, selected oil, and Span^®^ 80 (surfactant) were mixed at 80.0 ± 2.0 °C to prepare the oily phase. An aqueous phase, containing Tween^®^ 80 in Milli-Q water, was heated at the same temperature. The hot aqueous phase was then added dropwise to the oily phase while maintaining continuous magnetic stirring at 2000 rpm for 10 min to form a primary emulsion. The primary emulsion was then homogenized at 14,000 rpm for 5 min using a T25 digital Ultra-Turrax (IKA, Wilmington, NC, USA) to further reduce the GS. The macroemulsion obtained was further subjected to probe sonication at 40% amplitude with a 3 mm stepped microtip at 500 watts power supply and 115 volts for 10 min with a 10 s pulse ON and 10 s pulse OFF using Sonic Dismembrator (Model: FB-120, Fisherbrand^TM^, Waltham, MA, USA) to form NE.

#### 2.3.2. Preparation of the Mucoadhesive KZ-NE (KZ-NEC)

The NEC formulations were prepared following the same procedure described for NEs, with a slight modification. The total volume of Milli-Q water was divided into two equal portions: one portion was used to dissolve the mucoadhesive agent (Carbopol^®^ 940 NF) to form its aqueous solution, while the other portion was utilized to prepare the aqueous phase as previously described.

#### 2.3.3. Preparation of KZ Solution (KZ-S)

The drug solution was prepared by adding KZ (1.0% *w*/*v*) to ethanol (30% *v*/*v* in water) and used as a control for in vitro antifungal susceptibility testing [22].

#### 2.3.4. Control Formulation (Cream, KZ-C)

KZ cream is readily available in Egypt (kafrelsheikh, kafrelsheikh, Egypt), sold under the brand name Nizoral^®^ (2% *w*/*w*).

#### 2.3.5. Characterization of KZ-NEs and KZ-NEC

##### Measurement of Globule Size, Polydispersity Index, and Zeta Potential

Globule size (GS) and polydispersity index (PDI) of the prepared formulations were measured by photon correlation spectroscopy using a Zetasizer (Nano ZS Zen3600, Malvern Panalytical Inc., Westborough, MA, USA) at 25 °C. The measurements were performed in disposable solvent-resistant micro cuvettes (ZEN0040, Malvern Panalytical Inc., Westborough, MA, USA), which were pre-flushed out with 0.22 μm filtered Milli-Q water before sample loading to eliminate any dust/particulates in the cuvette before performing any measurements [23]. Samples were diluted 100 times with filtered Milli-Q water. GS and PDI were measured using a helium-neon laser, and data were evaluated based on volume distribution. Zeta potential (ZP) was measured for the same diluted samples. All measurements were conducted in triplicate.

##### KZ Content

For drug content analysis, the prepared formulations (100 μL) were extracted in an organic solvent (methanol, 900 μL). The mixture was centrifuged (Fisherbrand^TM^, Waltham, MA, USA) for 20 min at 13,000 rpm, and the supernatant was analyzed for KZ content, using the analytical method described above, following appropriate dilution with methanol.

##### pH Measurement

The pH of the prepared KZ-NE formulations was measured using a METTLER TOLEDO pH meter (FiveEasy™, Columbus, OH, USA). The pH meter was calibrated with pH Buffer Kit with known pH values (Orion™ Standard All-in-One™ pH Buffer Kit, 4.01, 7.00, and 10.01, Thermo Fisher Scientific, Chelmsford, MA, USA). The pH meter was calibrated to compensate for any changes that may have occurred in the electrode and to ensure that the pH readings remain accurate and reproducible. All pH measurements were performed in triplicate.

##### Viscosity Measurement

The viscosity of NEs was determined using a Brookfield cone and plate viscometer (LV-DV-II+Pro Viscometer, Middleboro, MA, USA). The gap between the cone and plate was adjusted before the measurement. Then, the formulation (0.5 mL) was placed in the cup plate. The sample inside the cup was maintained at 25 °C using a circulating water bath. A CPE 52 spindle was operated at 10 rpm, and the viscosity was recorded in standalone mode.

#### 2.3.6. In Vitro Release Testing

Phosphate buffer (pH 7.4) and Tween^®^ 20 (2% *v*/*v* to maintain sink condition) mixture was used as the receiver medium for in vitro release testing and ex vivo permeation and deposition testing [24]. KZ release studies were performed through a vertical Franz cell apparatus (Logan Instruments Corp., Somerset, NJ, USA). Cellulose membrane (thickness: 0.44 mm, pore size: 12–14,000 Da) was fixed between the two half-cells of the diffusion cell with an active diffusion area of 0.636 cm^2^. Cellulose dialysis membrane, which is chemically inert and lacks functional groups capable of interacting with KZ, was deliberately selected to minimize drug–membrane binding. A fixed volume of formulation (200 μL) or control (100 mg) was applied to the donor compartment above the cellulose dialysis membrane. This volume provided an infinite dose condition, as the applied amount of KZ greatly exceeded its solubility in the receptor medium. The receiver chamber was filled with release medium (5 mL) and kept under continuous magnetic stirring at 32.0 ± 0.5 °C with the aid of a circulating water bath. Aliquots (0.5 mL) were collected from the receiver chamber and replenished with an equivalent volume (0.5 mL) of the freshly prepared release medium to maintain sink conditions at pre-determined time points. Samples were quantified for KZ using the analytical method described above. he release data were evaluated for their goodness of fit to zero-order, first-order, Higuchi, and Korsmeyer–Peppas kinetic models using DDSolver, a free Excel add-in designed for drug release analysis (Microsoft Excel 2019, Microsoft Corporation, Redmond, WA, USA).

#### 2.3.7. Ex Vivo Permeation

The rats were housed with unrestricted access to food and water and were euthanized under ether anesthesia. The abdominal area was then shaved using a depilatory agent. The skin was surgically excised, and the subcutaneous tissue was carefully separated. The dermal side was cleaned with isopropyl alcohol to remove residual fat. Full-thickness skin samples were rinsed with isotonic phosphate-buffered saline (IPBS), wrapped in aluminum foil, and stored at −20 °C until further use [18]. Test and control formulations were selected for ex vivo permeation testing, using a vertical Franz diffusion apparatus (Logan Instruments Corp., Somerset, NJ, USA). Before the study, the rat skin samples were thawed at room temperature. Then, the skin was mounted between the two half-cells of the diffusion cell with the stratum corneum facing the donor chamber, which contains either the test (200 μL) or the control formulation (100 mg). The formulations were placed in the donor chamber above the skin using a syringe. The KZ content of the receiver chamber was maintained under continuous magnetic stirring at 32.0 ± 0.5 °C with the aid of a circulating water bath. Samples (0.5 mL) were withdrawn at pre-determined time points from the receiver chambers within six hours and replenished with an equivalent volume of the receiver medium. The amount of KZ in samples was quantified using the UFLC method described above.

The cumulative amount of KZ permeated (Q_n_), steady-state flux (J_ss_), and transdermal permeability coefficient (P_eff_) were calculated to study the ex vivo permeation of KZ across rat skin. The analysis for all the samples was conducted in triplicate.

- Q_n_ was calculated using the following equation:

$$Q_{n}=V_{r}C_{r(n)}+\sum_{x=1}^{x=n}V_{s(x-1)}C_{r(x-1)}$$

where n represents the sampling time point, V_r_ represents the volume of the receiver chamber (mL), V_s_ represents the volume of the sample withdrawn from the receiver chamber at the nth time point (mL), and C_r(n)_ is the concentration of KZ in the receiver chamber at the nth time point. The skin permeation rate (dQ/dt) was determined from the slope of the plot of Q_n_ versus time.

- J_ss_ of KZ was calculated using the following equation:

J_ss_ = (dQ/dt)/A

where Q is the amount of KZ transported through the skin.

- The transdermal permeability coefficient was calculated by the following equation:

P_eff_ = J_ss_/C_0_

where C_0_ is the initial donor concentration for KZ.

#### 2.3.8. Ex Vivo Deposition Study

Following the ex vivo permeation experiment, the skin was removed from the diffusion cell and washed with methanol-soaked cotton swabs (Q-tips^®^) to remove any superficially unabsorbed KZ. Then, the effective skin diffusion area was weighed, cut into small pieces, and dissolved in methanol. The skin was then homogenized in methanol for 2 h to extract the drug. The resulting mixture was centrifuged for 15 min at 13,000 rpm [24]. The supernatant was then collected after centrifugation and injected into UFLC to quantify KZ within skin tissue homogenate by applying the following equation:

Q_d_ = Q_s_/W_s_

where Q_d_ represents the amount of KZ deposited per milligram of skin; Q_s_ denotes the quantity of KZ measured within the effective permeation area; and W_s_ is the weight of that effective permeation area.

#### 2.3.9. In Vivo Studies

The Dermatokinetics of the optimized test and control formulations were performed in male Wistar albino rats, after a single topical application under fasting conditions. Male Wistar albino rats were selected for the in vivo and dermatokinetic studies to minimize physiological variability associated with hormonal fluctuations observed during the estrous cycle in female rats. Sex-dependent hormonal changes have been well documented to influence skin permeability, drug absorption, and pharmacokinetic parameters, which may confound comparative formulation-based evaluations. The use of male animals, therefore, allowed for improved reproducibility, reduced inter-animal variability, and clearer interpretation of formulation-dependent differences in transdermal drug delivery.

Rats were maintained under standard laboratory conditions, housed in polypropylene cages with unrestricted access to a standard laboratory diet and water for 7 days before starting the study. Before the study, rats were carefully examined for any skin abnormalities, and those exhibiting lesions or acne-like conditions were excluded from the experiment. Approximately 10 ± 1 cm^2^ of skin on the dorsal side of each rat was shaved, and the animals were subjected to overnight fasting before the study. The animals were randomly allocated into three groups, with each group comprising six healthy male Wistar albino rats. Groups I was treated with the commercial KZ cream (100 mg) as the control formulation. Groups II and III were treated with the optimized KZ-NE and lead KZ-NEC formulations (200 μL), respectively. Blood samples (0.5 mL) were collected at predetermined time intervals (0, 0.5, 1, 2, 3, 4, 6, 8, 10, 12, and 24 h) through the marginal ear vein following ether anesthesia. Serum was separated by centrifugation at 10,000 rpm for 10 min and stored at −20 °C until analysis.

#### 2.3.10. Quantification of KZ During In Vivo Studies

The KZ content in rat serum was quantified using a previously reported HPLC method [25]. Briefly, 100 μL of serum was mixed with 100 μL of piperine solution (1 μg/mL) as an internal standard and vortexed for 3 min (5 min, 2000 rpm, Vortex-Genie^®^ 2, Scientific Industries, Inc., Bohemia, NY, USA). Subsequently, 300 μL of acetonitrile was added as a protein-precipitating agent, and the mixture was vortexed for 5 min. The sample was then centrifuged at 10,000 rpm for 10 min, and the resulting supernatant was collected. The supernatant was evaporated in a vacuum oven at 50 °C, and the dried residue was reconstituted in 100 μL of the mobile phase. A 20 μL aliquot of the reconstituted solution was injected into the HPLC system for analysis. The analysis was carried out using A Dionex UltiMate 3000 HPLC (Thermo Scientific, Dionex, Sunnyvale, CA, USA). A mixture of KH_2_PO_4_ (25 mM, pH 4.5) and acetonitrile at a ratio of 50:50 *v*/*v* was used as mobile phase at a flow rate of 1.0 mL/min, using a Waters Symmetry^®^ C18 column (5 μm, 150 × 4.6 mm, Waters, Milford, CA, USA). The detection wavelength was set to 230 nm. All sample handling was performed under light-protected conditions. The peak serum concentration (C_max_) and time to reach peak concentration (t_max_) were determined.

#### 2.3.11. Skin Irritation Studies

Skin irritation studies of both lead and control formulations were performed in male Wistar albino rats using the Draize patch method [26]. The rats were divided into five groups, each consisting of four animals weighing between 190 and 250 g. The hair on the rats’ dorsal region was clipped 24 h before the experiment, and the skin was examined for any abnormalities. The formulations were applied to a 5 ± 1 cm^2^ area on the dorsal surface of the rats as follows: Group I served as the negative control; Group II received the optimized KZ-NE (200 μL); Group III received KZ-NEC (200 μL); Group IV was treated with the marketed cream formulation (100 mg); and Group V received formalin solution (200 μL, 0.8% *w*/*v*). Changes in skin color, morphology, and signs of erythema and edema were monitored for 24 h. The application sites were evaluated against control using a visual scoring scale [18].

#### 2.3.12. In Vitro Antifungal Activity

The antifungal activity of KZ from the test and control formulations was evaluated against *Candida albicans* cultures, a common opportunistic human fungus, using the cup plate method. *Candida albicans* MTCC 227 cultures were grown on 2% Sabouraud agar and maintained on slants at 5 ± 3 °C. Forty-eight hours before the assay, cultures were transferred to fresh slant agar and incubated at 32 °C. Microorganisms were suspended in 0.9% *w*/*v* normal saline to achieve a concentration of approximately 10^6^ cells/mL. The colony-forming units (CFU/mL) in each suspension were determined using the spread plate technique. An appropriate volume of the diluted inoculum (initial CFU ≈ 10^6^/mL) was incorporated into the melted Sabouraud agar to achieve approximately 100 CFU per 30 mL of medium. The medium was then poured into sterile Petri plates (30 mL per plate) and allowed to solidify. Four wells (1 cm^2^) were bored into each plate using a stainless-steel cylinder. Then, each well was filled with a measured volume/weight of the test (KZ-NE, KZ-NEC), negative control (placebo NE and NEC), or positive control (KZ-S, KZ-C) formulations using a sterile micropipette, ensuring no overflow and avoiding air bubbles. The plates were then incubated at 32 °C for 48 h to allow fungal growth and diffusion of the formulations. Following incubation, the antifungal activity was assessed by measuring the diameter of the zones of inhibition surrounding each well. Measurements were taken in millimeters using a calibrated digital Vernier caliper, ensuring accuracy by recording the average of two perpendicular readings for each zone, and this value was used as an indicator of antifungal activity.

#### 2.3.13. Physicochemical Stability Studies

A stability study for the lead formulation was conducted under refrigerated (5 ± 3 °C), room-temperature (30 ± 2 °C), and accelerated (40 ± 2 °C) storage conditions. The formulation was assessed at predetermined intervals for signs of physical instability, including cracking, changes in color, particle size, PDI, zeta potential, pH, viscosity, and drug content.

#### 2.3.14. Statistical Analysis

A minimum of three independent measurements have been performed, and the values of the data have been represented as mean ± standard deviation (SD). Statistical comparison was performed using SPSS (IBM SPSS Statistics software, SPSS 28, Armonk, NY, USA). Differences were deemed statistically significant when the *p*-value was less than 0.05.

## 3. Results and Discussion

### 3.1. Screening of Oil Excipients

Formulating nanoemulsions (NEs) with oils that have low drug-solubilizing capacity may require incorporating a larger quantity of oil to achieve the desired drug dose. However, increasing the oil content also necessitates higher concentrations of surfactant and/or cosurfactant to maintain proper solubilization. This can potentially exceed the maximum allowable daily exposure limits for these inactive ingredients [27]. Drug solubility in oil is a significant criterion for the selection of the oily phase. Lipophilic drugs with poor aqueous solubility, such as KZ, are preferably solubilized in O/W emulsions. Drug loading within NE formulations is a very critical design aspect in the formulation development of O/W NEs for poorly water-soluble drugs. Drug loading is mainly dependent on the drug solubility in various formulation ingredients. However, there is a risk of drug precipitation if the surfactant or cosurfactant is the main contributor to drug solubilization within the NE formulation since the dilution of NE could lower the solvent capacity of both surfactant and cosurfactant [14,27]. Therefore, drug solubility in the selected oil phase must be carefully considered when designing a stable NE system for topical delivery. Ensuring adequate solubility in the oil helps maintain the NE’s ability to undergo monophasic dilution upon contact with water, thereby minimizing the risk of drug precipitation in any aqueous environment [27,28,29]. The solubility of KZ in different oils was visually examined, as shown in Table 3. KZ showed solubility in oleic acid, soybean oil, olive oil, and castor oil, compared with the other screened oils. Thus, these four oils were the candidates for the saturation solubility studies.

### 3.2. Saturation Solubility Studies

Saturation solubility studies were conducted to quantify the maximum amount of KZ that could dissolve in the four candidate oils. The results showed that 205.4 ± 3.5, 95.7 ± 1.8, 11.2 ± 0.7, and 3.2 ± 0.4 mg of KZ were soluble in 1.0 g of oleic acid, castor oil, soybean oil, and olive oil, respectively (mean ± SD, n = 3). These findings indicate that KZ exhibits the highest solubility in oleic acid compared with the other tested oils. Accordingly, oleic acid was selected as the oily phase for the preparation of the KZ-NE formulations.

### 3.3. Factorial Design

A full factorial design was applied to optimize the composition of KZ-NE formulations in this study to provide better insight into how the critical properties of KZ-NE are affected by varying the quantities of different ingredients in the presence of KZ. The design of experiments allows investigating the effect of every variable (including secondary factors) and the interactions between independent variables with a reduced trial number (8). Table 1 and Table 2 show the independent and dependent variables with their coded levels of the full factorial design of this study, with the responses of all eight prepared KZ-NE formulations.

### 3.4. Statistical Analysis of GS

A wide range of GS was obtained for KZ-NE from batches prepared by varying the amounts of oleic acid, Span^®^ 80, and Tween^®^ 80. After data analysis using DesignExpert^®^ software, it was obvious that all independent variables significantly affect the GS of KZ-NEs. GS obtained from the eight runs ranged from 96.7 ± 2.9 to 171.9 ± 5.8 nm. Analysis of variance (ANOVA) indicated that the selected factorial model was highly significant (*p* < 0.0001). The correlation statistics supporting the fit of the factorial model for the GS response are presented in Table 4. The model’s F-value of 173.33 further confirmed its statistical significance. A, B, and C were the only significant model terms. The predicted R^2^ value showed good agreement with the adjusted R^2^ value, with a difference of less than 0.2, indicating strong predictive capability of the model. The following equation represents the final equation to predict GS in terms of coded factors:

GS = 132.11 + 17.76 × A + 12.61 × B − 7.39 × C

The mean GS of KZ-NEs was significantly increased with the increase in oleic acid (A) and Span^®^ 80 concentrations (B) and the decrease in Tween^®^ 80 concentrations (C). The increase in GS with higher amounts of oleic acid could be due to insufficient surfactant amount, required to form a large number of micelles to entrap the oil, which in turn is responsible for increasing the GS of the NE [30]. Moreover, the increase in the % of Span^®^ 80 in the surfactant mixture decreased the hydrophilic–lipophilic balance (HLB) value of the surfactant mixture, which increased GS significantly (*p* < 0.05). At the same time, increasing total surfactant (B + C) concentration significantly reduced the effect of oleic acid on GS, with GS mainly governed by the total surfactant concentration. This could be due to a higher concentration of surfactant, which aids in stabilizing small oil globules and prevents these oil globules from coalescing into larger globules [30,31].

### 3.5. Statistical Analysis of ZP

It was obvious that only one independent factor significantly affected the ZP of KZ-NEs. ZP obtained from the eight runs ranged from −23.5 ± 0.9 to −28.5 ± 0.3 mV. ANOVA demonstrated that the proposed design was statistically significant (*p* < 0.0008). The correlation statistics supporting the fit of the factorial model for the ZP response are presented in Table 4. The model’s F-value of 38.53 further confirmed its significance, with oleic acid identified as the only significant model term. The predicted R^2^ value showed good agreement with the adjusted R^2^ value, with a difference of less than 0.2, indicating strong predictive capability of the model. The following equation represents the final equation to predict ZP in terms of coded factors:

ZP = −26.19 − 1.69 × A

The mean ZP of KZ-NEs was significantly increased (i.e., an increase in absolute value) with the increase in oleic acid concentration (A). Since ZP sign and magnitude are related to the net charge type and density, respectively, displayed on the oil globule surface, the results suggest that the accumulation of more negative charges on the surface of the NE globules occurs with increasing the oleic acid content. Comparable findings have also been documented in several previously published studies [32,33]. Oleic acid molecules carry ionizable carboxylic acid groups and, thus, increasing the oleic acid concentration in the NE might affect the ZP magnitude. However, this is unlikely to be a conclusive explanation for the increase in ZP negativity (i.e., increase in absolute value) with increasing the concentration of oleic acid. The size of the dispersed oil globule has been reported to positively influence its ZP. In other words, the increase in GS is usually associated with an increase in the absolute value of ZP [33,34]. This could be due to the fact that as the GS increases, so does its net negative charge, resulting in a stronger electric field and, as a result, a higher ZP value [33]. This explanation appears to be consistent with the findings in Table 2, which demonstrate that as the volume fraction of the dispersed phase increases, both GS and the absolute value of ZP increase. Therefore, GS, ZP, and volume fraction of the dispersed phase of the O/W emulsions are correlated.

### 3.6. Optimization

Based on the literature review, the ideal topical NE preparation should have a GS of <500 nm, a PDI value of 0.1–0.3, and a ZP of ±30 mV [35]. After analyzing the responses and establishing reliable regression models, an optimization step was carried out to determine the optimal factor levels. The criteria for the variables and responses used in this optimization process are summarized in Table 5. To obtain the desired outcomes within a 95% confidence interval (CI), the software generated a single optimal solution, which closely matched the composition of formulation F. This solution is graphically represented by the cubes shown in Figure S1. A validation trial was conducted in triplicate to compare the actual GS and ZP values against their predicted values, as shown in Table 6. The mean of actual GS and ZP values was found to be within a 95% CI of the predicted values.

### 3.7. Preparation of KZ-NE and KZ-NEC

The type and concentration of excipients used during formulation development are critical factors that directly influence both the safety and overall suitability of the final product. Tween^®^ 80 was selected since hydrophilic surfactants with a hydrophilic–lipophilic balance (HLB) value >10 were reported to produce uniform O/W NEs [30]. In addition, nonionic surfactants such as Tween^®^ 80 and Span^®^ 80 are reported to have lower irritation potential and toxicity compared to ionic surfactants [30,36]. Moreover, all formulation excipients were evaluated at concentrations falling within the FDA-approved inactive ingredient limits for topical drug products, as listed in Table 7.

Carbomer copolymer types A, B, and C, and Carbomer homopolymer types A, B, and C have been used in many FDA-approved topical products. Amongst these polymers, Carbopol^®^ 940 NF is a highly efficient thickener used for preparing clear aqueous and hydroalcoholic gels. It produces the highest viscosity (40,000–60,000 cP at 0.5% weight and a pH of 7.5) and can be used between 0.5% and 3.0% *w*/*w* in topical formulations. Accordingly, Carbopol^®^ 940 NF was added to convert the optimized KZ-NE (F2) formulation to the KZ-NEC formulation. Carbopol^®^ 940 NF offers the benefit of remaining in a liquid state under acidic pH conditions. When the pH rises above 5.5, Carbopol^®^ 940 NF transitions from a sol to a gel, forming a viscoelastic gel [37]. Therefore, to enhance the adherence of the formulations to the skin surface, Carbopol^®^ 940 was chosen for this study. Carbopol^®^ 940 NF concentration was optimized based on the viscosity of the formulations after testing four different concentrations. The composition of all prepared KZ-NE and KZ-NEC formulations is presented in Table 8. All prepared NE formulations were loaded with 1% *w*/*v* of KZ.

### 3.8. Viscosity Measurement

**Figure 1:**
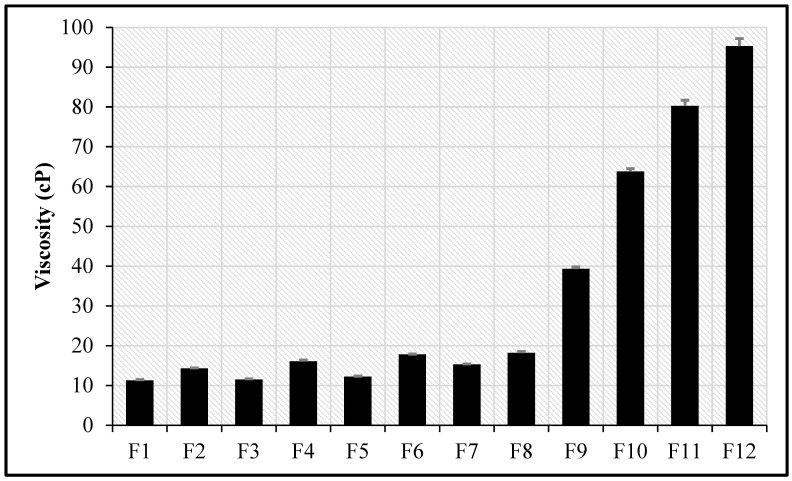
The viscosity of different KZ-NEs and KZ-NEC formulations (mean ± SD, n = 3).

The rheological behavior of dermal drug delivery systems represents a fundamental parameter in formulation development. Viscosity not only determines critical product performance characteristics—such as spreadability and the sensory profile during application—but also plays a significant role in modulating both the rate and extent of transdermal penetration of incorporated active pharmaceutical ingredients [38]. Drug penetration has been reported to decrease with increasing the viscosity of different dermal formulations [38]. The viscosity values for all formulations are depicted in Figure 1. The viscosity values ranged from 11.3 ± 0.2 to 18.2 ± 0.3 cP. The viscosity of the optimized KZ-NE formulation was significantly increased (*p* < 0.05), as expected, after the incorporation of Carbopol^®^ 940 NF within the formulation (F9–F12). The viscosity of Carbopol^®^ 940 NF containing formulations (F9–F12) ranged from 39.3 ± 0.5 to 95.3 ± 1.9 cP. This increase was aligned with expectations based on the polymer’s rheological behavior.

The initial low viscosity ensures ease of application, which is crucial for patient compliance. For in situ gelling dosage forms applied to the skin, a low initial viscosity (solution state) is optimal for easy application, followed by a significant increase in viscosity (gel state) after application to ensure prolonged residence time. The formulation should be a free-flowing liquid, typically with a viscosity in the range of 5 to 1000 cP, to allow for simple and convenient application to the skin before gel formation on the skin. The high viscosity after gelation provides good bioadhesion, increasing the formulation’s residence time on the skin and thus enhancing drug permeation. However, increased viscosity can slightly hinder the rate of drug diffusion out of the formulation [38,39,40]. Therefore, the authors decided to continue with the lowest Carbopol^®^ 940 NF concentration containing formulation as the lead formulation (F9).

### 3.9. Measurement of GS, PDI, and ZP

GS, PDI, and ZP are depicted in Table 2 and Figure S2. A homogeneous system with GS ranging from 96.7 ± 2.9 to 171.9 ± 5.8 nm, with a surface charge from −23.5 ± 0.9 to −28.5 ± 0.3 mV, and PDI from 0.13 ± 0.02 to 0.19 ± 0.03 was obtained. Nanosize could greatly affect the amount and the depth of drug deposition through skin layers [15]. NEs with a GS less than 200 nm are more preferred for dermal drug delivery [41]. PDI values reveal the width of the GS distribution, with lower values indicating greater uniformity. PDI of less than 0.3 is generally indicative of a well-dispersed nanoparticulate system characterized by a narrow and homogeneous GS distribution [42].

A zeta potential (ZP) exceeding ±30 mV is generally indicative of good electrostatic stabilization of nanoemulsions, whereas values approaching ±60 mV reflect exceptionally high stability due to strong repulsive forces between dispersed droplets [43]. In contrast, ZP values above ±20 provide only short-term NE stability, while a −5 to +5 mV range is associated with rapid aggregation of the dispersed oil droplets [43]. Based on this information, the F2 formulation was a good candidate according to the software.

Carbopol^®^ 940 NF addition changed ZP significantly from −28.5 ± 0.3 for the optimized NE (F2) to −38.2 ± 0.8 for the F9 formulation, probably due to the numerous negatively charged carboxylic acid groups along the polymer backbone. The significant change in ZP suggests that Carbopol^®^ 940 NF is adsorbed on the surface of the oil globules [20]. The significant increase (*p* < 0.05) in GS from 141.6 ± 3.2 for the F2 formulation to 205.4 ± 5.2 for the mucoadhesive formulation (F9) also supports surface adsorption of Carbopol^®^ 940 NF [44]. Moreover, the PDI of the mucoadhesive formulation increased simultaneously with GS from 0.13 ± 0.02 for the F9 formulation to 0.28 ± 0.03.

### 3.10. Drug Content

According to the United States Pharmacopeia (USP), a variation of ±10% in drug content (assay) from the label claim is acceptable to accommodate manufacturing variability and potential changes during shelf-life. Such variability is not expected to adversely affect the intended therapeutic outcome or the safety profile of the formulation. Drug content is therefore considered an important indicator of the chemical stability of nanodispersed systems, including NEs. In the present study, the drug content of the prepared KZ-NE formulations ranged from 97.5 ± 3.3% to 99.7 ± 1.3%, as shown in Figure S3. The lead Carbopol^®^ 940 NF containing formulations showed a similar drug content value to the optimized NE formulation.

### 3.11. pH Measurement

**Figure 2:**
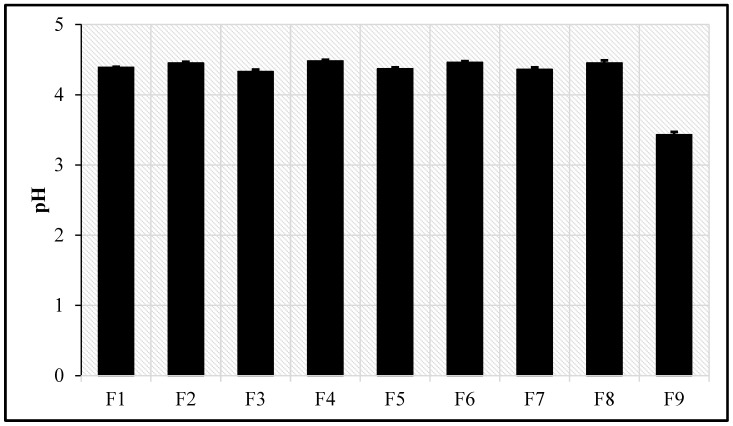
pH values for all prepared Ketoconazole nanoemulsions (mean ± SD, n = 3).

There is a high level of agreement that topically applied products should be acidified to a pH in the range of 4.0 to 6.0 [45]. The acidic pH of the skin has been identified as a regulatory factor in the maintenance of stratum corneum homeostasis and barrier permeability [45]. The pH values for all KZ-NE formulations are depicted in Figure 2. A narrow range of pH values was obtained for all prepared KZ-NE batches (4.33 ± 0.03–4.48 ± 0.02). It was observed that the pH of the optimized KZ-NE formulation (4.45 ± 0.02) was significantly (*p* < 0.05) decreased after the incorporation of Carbopol^®^ 940 NF (F9; 3.43 ± 0.04) within the formulation. This outcome could be due to the carboxylic acid groups of Carbopol^®^ 940 NF.

### 3.12. In Vitro Release Testing

**Figure 3:**
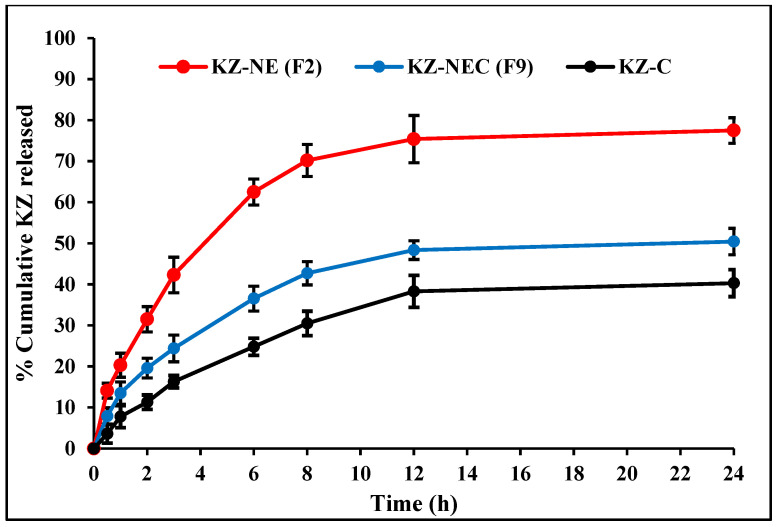
In vitro release of Ketoconazole from optimized KZ-NE, lead KZ-NEC, and marketed cream (Nizoral^®^) formulations (mean ± SD, n = 3).

In vitro release profiles of KZ from optimized KZ-NE, lead KZ-NEC, and marketed cream (Nizoral^®^) formulations were evaluated using a Franz diffusion cell-based in vitro release testing using a dialysis membrane. The cumulative % of KZ released from each formulation was plotted as a function of time. The in vitro drug release profiles are graphically illustrated in Figure 3. The cumulative released amount of KZ was in the following descending order: optimized NE (77.5 ± 3.1%), the mucoadhesive NEC (50.4 ± 3.2%), and the KZ cream (40.3 ± 3.3%) after 24 h. All tested formulations sustained the release of the loaded drug. The entrapment of KZ molecules within the oil globules, combined with the slow diffusion of the drug from the oil phase into the aqueous phase, likely contributed to the sustained-release behavior observed in both the NE and NEC formulations [23,46]. In addition, KZ is a highly lipophilic drug with a log *p* value of 4.74, having low aqueous solubility [47]. However, KZ release from the lead Carbopol^®^ 940 NF-containing NE formulation (F9) was slower than that from the optimized KZ-NE formulation (F2). This was expected as the lead NEC formulation showed higher viscosity compared to the optimized NE formulation. Moreover, the marketed cream formulation showed a slower KZ release compared to the optimized KZ-NE and lead KZ-NEC formulations. This is not surprising because creams are designed as semisolid emulsions that require the drug to diffuse through a complex base before reaching the skin.

Each of the three data sets was fitted to four conventional release models—zero-order, first-order, Higuchi, and Korsmeyer–Peppas—and regression analysis was carried out to evaluate the goodness of fit. The model exhibiting the highest coefficient of determination (R^2^) was considered the most appropriate for describing the release kinetics, as presented in Table 9. Model fitting indicated that the dissolution profiles of both developed formulations were best described by the Korsmeyer–Peppas kinetic model. The slope of the Korsmeyer–Peppas model, release exponent, indicates the mechanism of drug release from the developed formulation. The slope (n) of the optimized KZ-NE formulation and the marketed cream was 0.612 and 0.474, respectively, which indicated a non-Fickian (0.45 < n < 0.89) or anomalous drug release profile controlled by erosion and diffusion mechanisms. In contrast, the slope of the lead KZ-NEC formulation was 0.390, which indicated a Fickian (0.45 ≤ n) drug release profile controlled mainly by a diffusion mechanism. The sustained-release behavior commonly observed in O/W nanoemulsions can be attributed to the multistep release process of hydrophobic drugs starting from diffusion of the drug from oil globules into surfactant and then into the aqueous phase [14,23].

### 3.13. Ex Vivo Permeation and Deposition Testing

**Figure 4:**
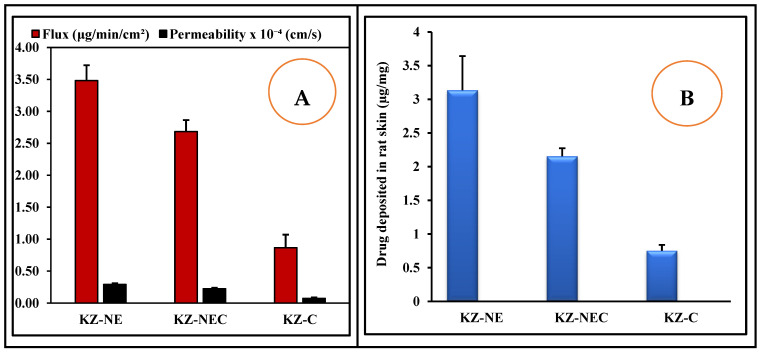
(**A**) Transdermal flux and permeability coefficients and (**B**) skin deposition results of Ketoconazole from optimized KZ-NE, KZ-NEC, and marketed cream formulations through rat skin (mean ± SD, n = 3).

Ex vivo skin permeation and drug deposition studies serve as valuable predictors of percutaneous absorption in humans, providing insight into both the rate and extent of transdermal drug delivery under controlled experimental conditions [48]. The ex vivo permeation profiles of all tested formulations are presented in Figure 4A,B. The flux of the KZ from the tested formulations was in the following descending order: KZ-NE (3.48 ± 0.22 μg/min/cm^2^), KZ-NEC (2.68 ± 0.16 μg/min/cm^2^), and marketed cream (KZ-C, 0.87 ± 0.12 μg/min/cm^2^). The transdermal permeability coefficients from KZ-NE and KZ-NEC formulations were 0.29 ± 0.02 and 0.22 ± 0.02 × 10^−4^ cm/min, respectively. The ex vivo transdermal flux and permeability coefficient of KZ from the optimized KZ-NE formulation were approximately four-fold greater than that achieved with the marketed cream formulation. Similarly, the ex vivo transdermal flux and permeability coefficient of KZ from the lead KZ-NEC formulation were approximately three-fold greater than that achieved with the marketed cream formulation. The reasons for the enhanced transdermal penetration from both NE-based formulations could be due to the following reasons: oleic acid has an inherent permeation-enhancing ability because it results in the formation of lacunae and perturbation of the intercellular lipid bilayers in the stratum corneum, consequently increasing drug penetration through this limiting and protective barrier [49,50]. In addition, surfactants can enhance drug permeation through the skin, which is due to their reversible binding to keratin filaments, which consequently leads to the disruption of corneocytes, thus altering the diffusion characteristics of the stratum corneum [49,51]. Moreover, small GS of the NE formulations provides a large surface area for drug transfer into the skin and a better chance to adhere to barrier membranes [52,53]. However, the ex vivo transdermal flux and permeability coefficient of KZ from the optimized KZ-NE formulation were significantly (*p* < 0.05) higher than those observed with its corresponding Carbopol^®^ 940 containing formulation (KZ-NEC). This outcome is likely due to the slower permeation of KZ caused by the increased viscosity of the formulation. Moreover, both NE-based formulations exhibited significantly higher skin deposition compared to the control formulations. This outcome could be due to the adsorption and fusion of oil globules with the skin.

### 3.14. In Vivo Studies

**Figure 5:**
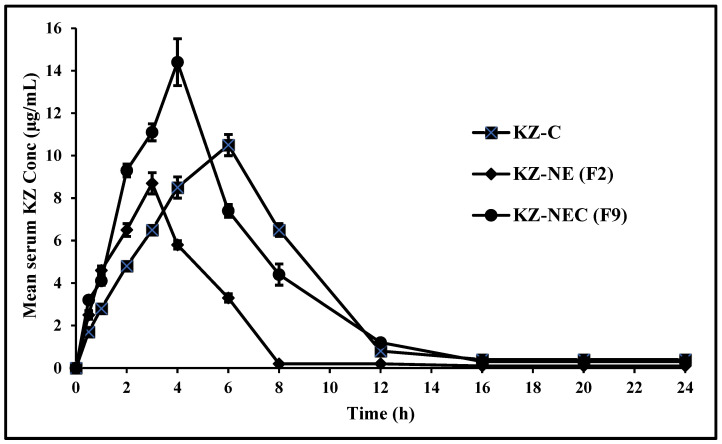
Mean serum concentration versus time profiles of Ketoconazole from optimized KZ-NE and lead KZ-NEC, and the marketed cream formulations after topical application in male albino rats (mean ± SD, n = 6).

Mean serum concentration versus time plots of KZ after topical application from all tested formulations are depicted in Figure 5. From the study’s findings, C_max_ of the lead KZ-NEC formulation (14.4 ± 1.1 μg/mL) was significantly (*p* < 0.05) higher compared with the marketed cream formulation (10.5 ± 0.5 μg/mL) and the optimized KZ-NE formulation (8.7 ± 0.5 μg/mL). In contrast, the T_max_ of the optimized KZ-NE formulation (3 h) was significantly (*p* < 0.05) lower compared with the lead KZ-NEC formulation (4 h) and the marketed cream formulation (6 h). Although the optimized KZ-NE formulation demonstrated higher ex vivo skin permeation parameters (flux: 3.48 ± 0.22 μg/min/cm^2^) compared with KZ-NEC (2.68 ± 0.16 μg/min/cm^2^), the in vivo dermatokinetic study showed a higher systemic exposure for KZ-NEC (C_max_ = 14.4 ± 1.1 μg/mL) than KZ-NE (C_max_ = 8.7 ± 0.5 μg/mL), indicating a lack of direct correlation between ex vivo permeation and in vivo absorption. These findings did not align with the ex vivo results of the optimized KZ-NE formulation, likely due to the short contact time available for drug absorption from this low-viscosity formulation. The improved absorption from the lead KZ-NEC formulation compared to the cream formulation is likely due to the oily phase within a cream forming a more occlusive layer on the skin surface, which requires more time to break down and soak in. This lipid-rich barrier slows the diffusion rate of KZ, resulting in a slower absorption time compared to the immediate, non-greasy penetration characteristic of gels.

### 3.15. Skin Irritation Studies

Skin irritation studies were carried out in rats, using the marketed cream as a control, formalin (0.8% *w*/*v*) serving as a positive control, and one group was untreated to serve as a negative control. The results of the study are presented in Table 10. Neither of the developed formulations caused any alterations in skin color or morphology during the 24 h visual observation period. In contrast, the marketed cream formulation exhibited signs of erythema after 24 h of topical application. Therefore, the findings indicate that both developed formulations are suitable for topical applications.

### 3.16. In Vitro Antifungal Activity

**Figure 6:**
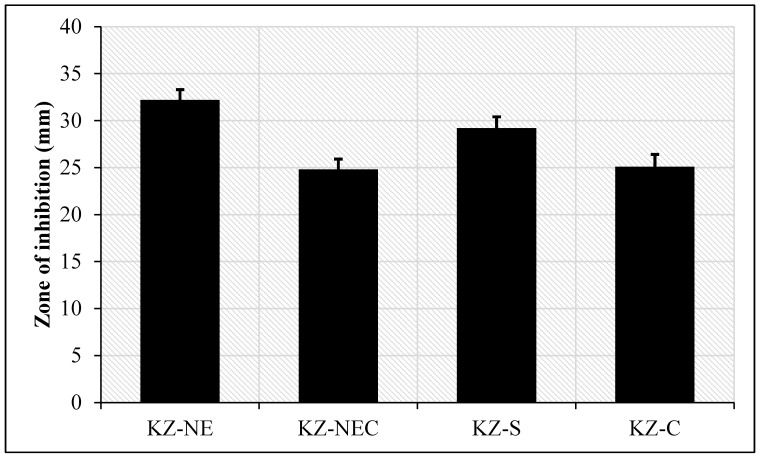
In vitro antifungal susceptibility testing of KZ-LE, KZ-NEC, KZ-S, and Ketoconazole cream against *Candida albicans* (mean ± SD, n = 4).

In vitro antifungal susceptibility testing showed that KZ demonstrated improved antifungal efficacy when incorporated into the NE and NEC formulations, as shown in Figure 6. An in-house prepared KZ solution (KZ-S) was also used in the current study because the cream formulation is too thick and the drug may not diffuse well during the test. The antifungal efficacy of the KZ-NE against *Candida albicans* was significantly (*p* < 0.05) greater than that of the marketed cream and KZ solution. The KZ-NE, with its large specific surface area, could interact easily with the fungal cell surface, contributing to its observed antifungal activity. This effect may also be attributed to the ability of the NE to approach the ergosterol composition of fungal hyphae [54]. In contrast, the antifungal efficacy of the KZ-NEC against *Candida albicans* was less than that of the KZ solution and the optimized KZ-NE formulation and approximately equal to the marketed cream. This was expected as the lead NEC formulation is more viscous than the KZ solution and the optimized KZ-NE formulation. It is worth mentioning that the negative control vehicles used in the study did not show activity against *Candida albicans.* In conclusion, the KZ-based NE and NEC formulations showed promising antifungal activity. Furthermore, KZ has an improved antifungal efficacy than the isolated drug when it is loaded inside NE. Since it would facilitate the penetration or interaction of KZ with the fungal cell surface. These findings align with previously reported results for sulconazole-loaded NEs, which similarly improved transdermal permeation and antifungal activity [54].

### 3.17. Physicochemical Stability

The physicochemical stability of the optimized KZ-NE and lead KZ-NEC formulations was determined by storage at refrigerated temperature, room temperature, and accelerated temperature conditions for 90 days (last-time point tested). The formulation did not show any phase separation, cracking, creaming, coalescence, or phase inversion upon visual inspection over the tested period. Moreover, only some statistically insignificant (*p* > 0.05) changes were observed in GS, ZP, PDI, pH, and drug content, as shown in Table 11.

The surfactant composition, globule size, and surface charge are key determinants of the physical stability of NEs, as they collectively influence interfacial behavior, droplet interactions, and resistance to aggregation. The collective influence of these three parameters ultimately governs the stability of nanoemulsions. An appropriate surfactant blend creates a resilient interfacial film between the two immiscible phases, enabling the dispersed phase to remain suspended as uniformly sized globules within the continuous medium [16]. The oil globules become elastic and can survive a high degree of tension during deformation. In addition, reducing the droplet size minimizes the influence of gravitational forces, allowing the dispersed oil globules to remain uniformly suspended within the continuous phase [55]. Moreover, numerous surface charge over the smaller oil globules generates strong interglobular repulsive forces, which effectively prevent oil droplet aggregation and contribute to maintaining dispersion stability [56]. The continuous Brownian motion of the oil globules within the dispersion medium makes these globules approach each other, and the globules become subjected to a strong repulsive force and finally move apart after the elastic collision [56]. This phenomenon improves kinetic stability [16]. Although high-molecular-weight surfactants such as Tween^®^ 80 and Span^®^ 80 typically impart a relatively low zeta potential, they nonetheless confer substantial stability to the NE through the combined effects of steric hindrance and the weak electrostatic repulsion generated between similarly charged globules [16]. According to Griffin’s hydrophilic–lipophilic balance (HLB) theory, combining surfactants to achieve a final HLB value in the range of 9–12 is generally suitable for formulating stable oil-in-water (O/W) nanoemulsions [57,58]. Thus, the 1:4 ratio of Tween^®^ 80 to Span^®^ 80, corresponding to a calculated HLB value of 12.9, is expected to confer excellent physical stability to the nanoemulsion system.

## 4. Conclusions

KZ-loaded NEs and NEC formulations were successfully developed and optimized, using Carbopol^®^ 940 NF as a gelling agent. The ex vivo skin permeation studies showed improved KZ skin permeability and flux when compared to the marketed cream. In addition, the C_max_ of the lead NEC formulation was significantly higher compared with the marketed cream formulation. However, the T_max_ was significantly lower. In addition, NE-based formulations were suitable for topical applications during the in vivo skin irritation testing. Furthermore, both developed formulations showed promising antifungal activity. Moreover, the formulations were physicochemically stable for 90 days (last-time point tested) at all tested storage conditions. Overall, the KZ-NE and KZ-NEC formulations prepared in this project are suitable for bioavailability enhancement of KZ through the skin. Thus, the NE combined with a gelling agent could serve as an efficient drug delivery platform for the different topical applications of KZ.

## Figures and Tables

**Table 1:** Independent and dependent factors with their coded levels of a full factorial design experiment.

Factors	Coded Levels	
Independent Variables	Level −1	Level +1
A: Oil (% *w*/*v*)	3.0	5.0
B: Span^®^ 80 (% *w*/*v*)	0.5	1.0
C: Tween^®^ 80 (% *w*/*v*)	2.0	3.0
Dependent Variables		
Y_1_: Globule size (GS)		
Y_2_: Zeta potential (ZP)		

**Table 2:** Full factorial design parameters that were obtained for various runs and experimental values of globule size and zeta potential for Ketoconazole-loaded nanoemulsions.

Run	Assigned Independent Variables			Actual Independent Variables			Response	
	A	B	C	Oil (Oleic Acid, % *w*/*v*)	Span^®^ 80 (% *w*/*v*)	Tween^®^ 80 (% *w*/*v*)	GS (nm)	ZP (mV)
1	−1	−1	−1	3	0.5	2	109.5 ± 0.5	−24.9 ± 0.9
2	+1	−1	−1	5	0.5	2	141.6 ± 3.2	−28.5 ± 0.3
3	−1	+1	−1	3	1	2	135.0 ± 4.9	−25.6 ± 1.4
4	+1	+1	−1	5	1	2	171.9 ± 5.8	−27.7 ± 0.5
5	−1	−1	+1	3	0.5	3	96.7 ± 2.9	−23.5 ± 0.9
6	+1	−1	+1	5	0.5	3	130.2 ± 3.8	−27.2 ± 1.0
7	−1	+1	+1	3	1	3	116.2 ± 3.4	−24.0 ± 0.7
8	+1	+1	+1	5	1	3	155.8 ± 7.2	−28.1 ± 0.9

**Table 3:** Screening of oils.

Oil	Solubility	Oil	Solubility
Soybean oil	(✓)	Capryol^TM^ 90	(x)
Cottonseed oil	(x)	Maisine^®^ CC	(x)
Sesame oil	(x)	Olive oil	(✓)
Castor oil	(✓)	Lauroglycol™ 90	(x)
Isopropyl myristate	(x)	Labrafac™ lipophile WL 1349	(x)
Maisine^®^ CC	(x)	Oleic acid	(✓)
Miglyol^®^ 829	(x)	Captex^®^ 200	(x)
Labrasol^®^	(x)	Captex^®^ 355 EP	(x)

(✓), (x): KZ is either dissolving or not dissolving in the oil, respectively.

**Table 4:** ANOVA of the regression coefficient of the fitted globule size and zeta potential equations.

Response	F-Value	*p*-Value	R^2^	Predicted R^2^	Adjusted R^2^	SD
Y_1_: GS	173.33	0.0001	0.992	0.971	0.987	2.81
Y_2_: ZP	38.53	0.0008	0.865	0.761	0.843	0.78

**Table 5:** Criteria of independent variables and responses for the optimization step.

Variable	Goal	Lower Limit	Upper Limit
Oleic acid	In range	3.0% *w*/*v*	5.0% *w*/*v*
Span^®^ 80	In range	0.5% *w*/*v*	0.5% *w*/*v*
Tween^®^ 80	In range	2.0% *w*/*v*	2.0% *w*/*v*
GS	In range	95.0 nm	500 nm
ZP	Target (−30.0 mV)	−27.0 mV	−32.0 mV

**Table 6:** Results of validation trials (n = 3).

Response	Predicted Value	Results of Validation Trials			95% CI (Low)	95% CI (High)
GS (nm)	144.7	148.3	139.6	147.9	133.5	154.1
ZP (mV)	−27.9	−28.7	−27.9	−28.4	−26.3	−29.3

**Table 7:** Inactive ingredients used in the preparation of Ketoconazole-loaded nanoemulsions as per the FDA database.

Inactive Ingredient	Topical Dosage Form	Maximum Potency per Unit Dose (% *w*/*w*)	Maximum Daily Exposure (MDE, mg)
Precirol^®^ ATO 5	NA		100 *
Oleic acid	Cream	25	
	Gel, Metered		88
	Solution	7.4	
Span^®^ 80	Cream	3.5	
	Cream, Augmented	0.2	
	Emulsion	2.5	
	Gel	1	
	Lotion	7	
	Ointment	NA	
	Spray	0.25	
Tween^®^ 80	Aerosol, Foam	0.98	
	Cream		4
	Emulsion	2.5	
	Gel	8.5	
	Lotion	15	
	Ointment	0.1	
Carbopol^®^ 940 NF	Cream		103
	Cream, Augmented		20
	Emulsion		6
	Gel		85
	Lotion		300
	Ointment, Augmented		23

*: Oral route. NA: Not applicable.

**Table 8:** The composition of different KZ-NEs and KZ-NEC formulations.

Formulation	Oleic Acid (% *w*/*v*)	Span^®^ 80 (% *w*/*v*)	Tween^®^ 80 (% *w*/*v*)	Ketoconazole (% *w*/*v*)	Carbopol^®^ 940 (% *w*/*v*)	Water up to (mL)
F1	3.0	0.5	2.0	1.0	-	10
F2	5.0	0.5	2.0	1.0	-	10
F3	3.0	1.0	2.0	1.0	-	10
F4	5.0	1.0	2.0	1.0	-	10
F5	3.0	0.5	3.0	1.0	-	10
F6	5.0	0.5	3.0	1.0	-	10
F7	3.0	1.0	3.0	1.0	-	10
F8	5.0	1.0	3.0	1.0	-	10
F9	5.0	0.5	2.0	1.0	0.5	10
F10	5.0	0.5	2.0	1.0	1.0	10
F11	5.0	0.5	2.0	1.0	2.0	10
F12	5.0	0.5	2.0	1.0	3.0	10

**Table 9:** Analysis of regression for the release kinetics of Ketoconazole from the optimized NE and the lead mucoadhesive NEC formulations (mean ± SD, n = 3).

Formulations	Zero-Order	First-Order	Higuchi	Korsmeyer–Peppas	
	*M*_0_ − *M* = *k.t*	*ln M* = *k.t*	*M*_0_ − *M* = *k.t*^1/2^	*log* (*M*_0_ − *M*) = *n log t* + *log k*	
	R^2^	R^2^	R^2^	R^2^	n
Optimized NE (F2)	0.1758	0.9076	0.8666	0.9987	0.612
Lead NEC (F9)	0.2968	0.6637	0.9091	0.9382	0.390
KZ-C	0.5662	0.7559	0.9437	0.9374	0.474

Where *M*_0_ represents the initial drug content at time *t*_0_, and *M* represents the drug content remaining at time *t*; zero-order model: % drug released vs. time; first-order model: amount drug remaining vs. time; Higuchi model: % drug released vs. square root of time; Korsmeyer–Peppas model: log % drug released vs. log time.

**Table 10:** Effect of the optimized KZ-NE and KZ-NEC formulations on male Wistar albino rat skin during 24 h of topical application (n = 4).

Group	Formulation	Erythema Score			Edema Score		
		6 h	12 h	24 h	6 h	12 h	24 h
I	Negative Control	0	0	0	0	0	0
II	KZ-NE	0	0	0	0	0	0
III	KZ-NEC	0	0	0	0	0	0
IV	KZ-C	0	0	1	0	0	0
V	Formalin (0.8% *w*/*v*)	0	1	2	1	2	3

Erythema scale: 0 = none, 1 = slight, and 2 = well-defined. Edema scale: 0 = none, 1 = slight, 2 = well-defined, and 3 = moderate.

**Table 11:** Globule size, polydispersity index, the zeta potential, pH, viscosity, and drug content of optimized KZ-NE (F2) and KZ-NEC (F9) formulations over 90 days of storage at 4, 25, and 40 °C (mean ± SD, n = 3).

Day		GS (nm)	PDI	ZP (mV)	pH	Viscosity (cP)	Drug Content (%)
		Storage at 5 ± 3 °C					
F2	0	138.5 ± 3.8	0.12 ± 0.01	−28.9 ± 0.4	4.45 ± 0.03	NA	98.6 ± 0.4
	90	139.1 ± 2.5	0.13 ± 0.01	−39.5 ± 0.4	4.42 ± 0.03		97.5 ± 2.7
		Storage at 30 ± 2 °C					
F2	0	139.9 ± 3.4	0.11 ± 0.02	−28.5 ± 0.5	4.43 ± 0.02	14.5 ± 0.1	98.8 ± 2.0
	90	142.4 ± 3.0	0.13 ± 0.03	−29.1 ± 0.7	4.44 ± 0.02	14.8 ± 0.2	99.3 ± 1.9
		Storage at 40 ± 2 °C					
F2	0	142.4 ± 5.2	0.12 ± 0.04	−31.2 ± 1.0	4.42 ± 0.03	NA	99.2 ± 1.2
	90	138.5 ± 2.5	0.12 ± 0.02	−30.1 ± 1.1	4.43 ± 0.03		97.9 ± 2.3
		Storage at 5 ± 3 °C					
F9	0	206.3 ± 2.9	0.29 ± 0.03	−38.2 ± 0.7	3.43 ± 0.02	NA	100.2 ± 3.8
	90	208.6 ± 4.1	0.28 ± 0.02	−37.2 ± 0.3	3.44 ± 0.02		99.2 ± 2.4
		Storage at 30 ± 2 °C					
F9	0	201.8 ± 2.9	0.27 ± 0.03	−39.4 ± 1.2	3.46 ± 0.03	38.8 ± 1.5	98.8 ± 1.8
	90	204.1 ± 4.7	0.29 ± 0.02	−38.8 ± 1.0	3.47 ± 0.02	40.2 ± 1.1	99.0 ± 1.7
		Storage at 40 ± 2 °C					
F9	0	207.3 ± 4.1	0.26 ± 0.04	−37.3 ± 1.5	3.38 ± 0.02	NA	99.5 ± 2.2
	90	203.5 ± 2.5	0.27 ± 0.03	−39.2 ± 0.5	3.45 ± 0.01		97.9 ± 2.5

NA: not applicable because viscosity is temperature-dependent.

## Data Availability

The original contributions presented in this study are included in the article and Supplementary Materials. Further inquiries can be directed to the corresponding author.

## Supplementary Materials

The following supporting information can be downloaded at: https://www.mdpi.com/article/10.3390/pharmaceutics18050612/s1, Figure S1: a contour plot represents the suggested solution by DesignExpert^®^ software; Figure S2: polydispersity index of all prepared Ketoconazole nanoemulsions (mean ± SD, n = 3); Figure S3: drug content for all prepared Ketoconazole nanoemulsions (mean ± SD, n = 3).
