# Supplementary material for: Ketoconazole-Loaded Mucoadhesive Nanoemulsions for the Better Management of Topical Fungal Infections: Optimization, In Vitro, Ex Vivo, and In Vivo Assessments
Source: Pharmaceutics. 2026 May 17;18(5):612. doi: 10.3390/pharmaceutics18050612 (PMC13210714; doi:10.3390/pharmaceutics18050612)
Supplement: Supplementary file 1 [file pharmaceutics-18-00612-s001.zip › pharmaceutics-4257753-supplementary.pdf]

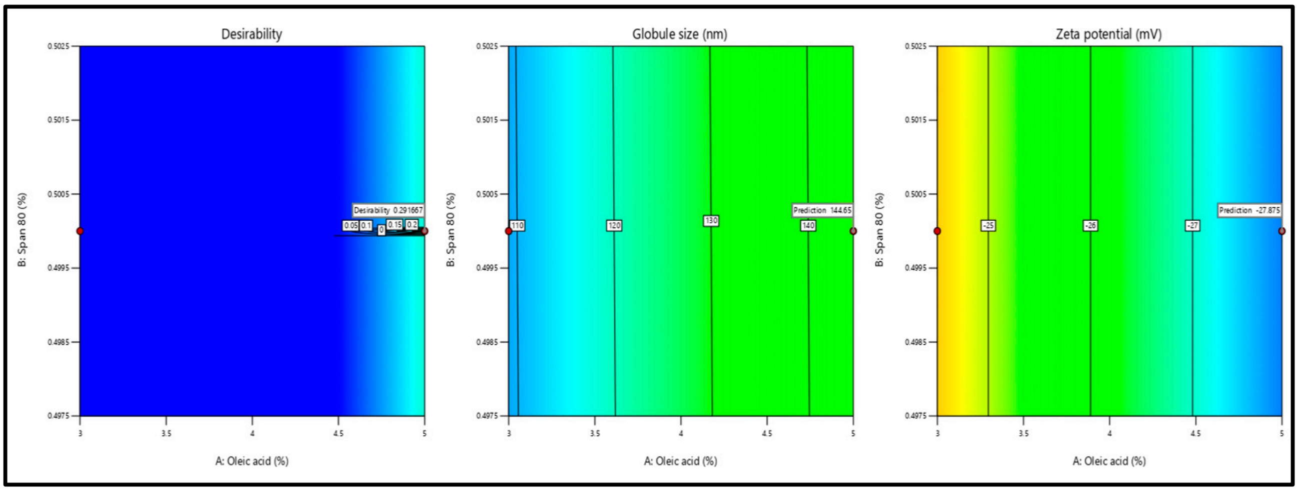

Figure S1. A contour plot represents the suggested solution by DesignExpert® software.

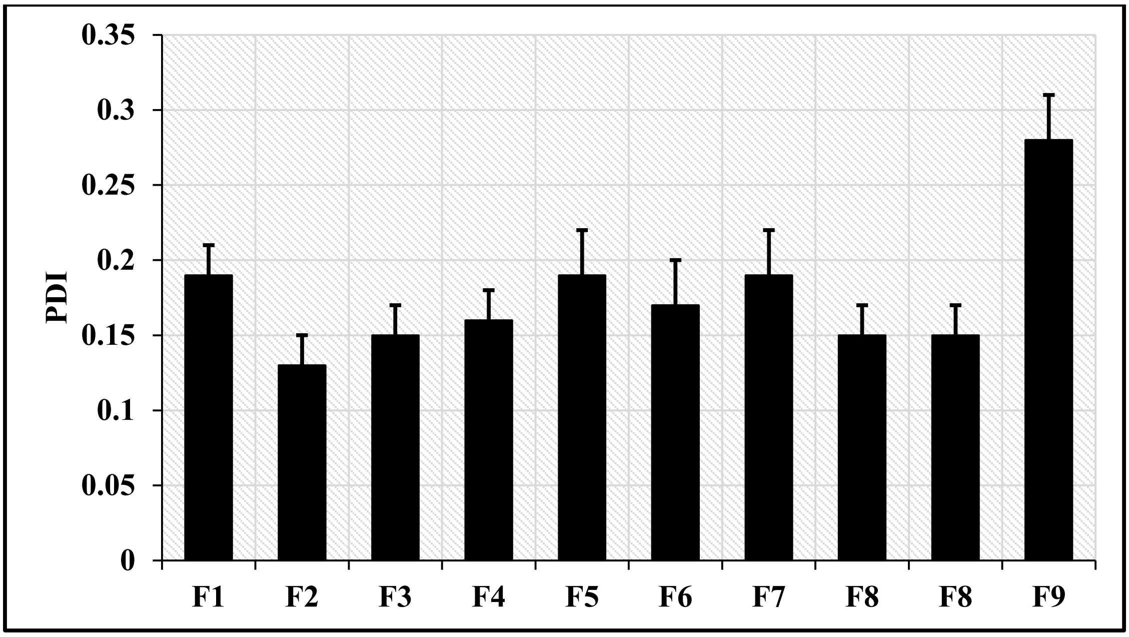

Figure S2. Polydispersity index of all prepared ketoconazole nanoemulsions (mean±SD, n=3).

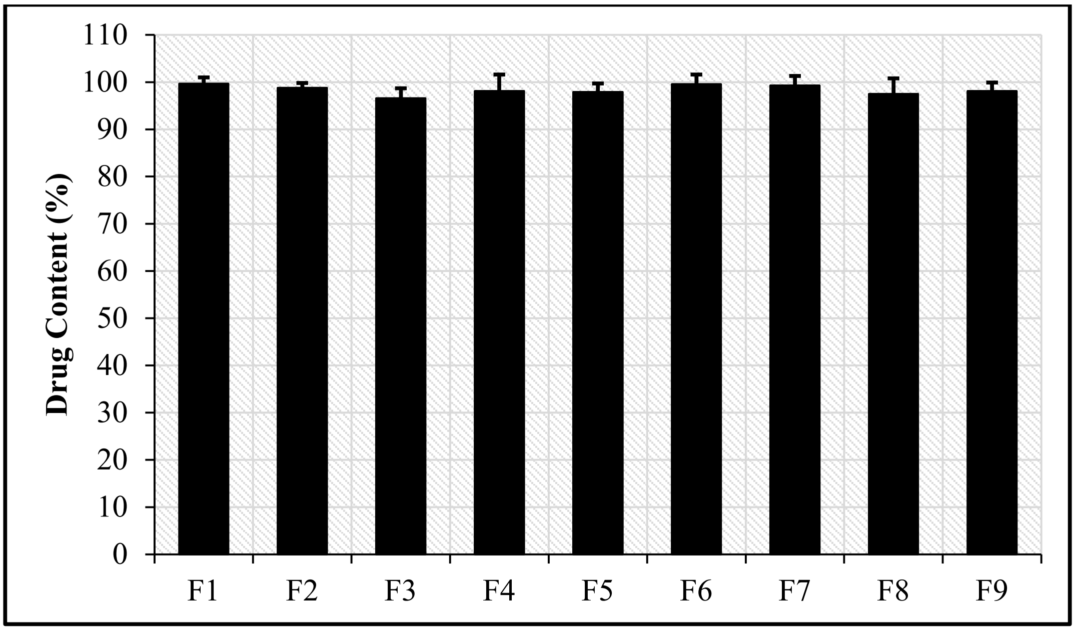

Figure S3. Drug content for all prepared ketoconazole nanoemulsions (mean±SD, n=3).
